# Biosensing Technologies for Detecting *Legionella* in Environmental Samples: A Systematic Review

**DOI:** 10.3390/microorganisms12091855

**Published:** 2024-09-06

**Authors:** Giuseppe Andrea Screpis, Andrea Aleo, Natalia Privitera, Giuseppe Emanuele Capuano, Roberta Farina, Domenico Corso, Sebania Libertino, Maria Anna Coniglio

**Affiliations:** 1 Department of Medical and Surgical Sciences and Advanced Technologies “G.F. Ingrassia”, University of Catania, Via S. Sofia 87, 95123 Catania, Italy; giuseppescrepis@hotmail.it (G.A.S.); andre.aleo93@gmail.com (A.A.); privitera.ntl@gmail.com (N.P.); ma.coniglio@unict.it (M.A.C.); 2 Institute for Microelectronics and Microsystems (CNR—IMM), HQ, National Research Council of Italy, VIII Street Z.I., 5, 95121 Catania, Italy; domenico.corso@imm.cnr.it (D.C.); sebania.libertino@cnr.it (S.L.); 3 Department of Chemical Sciences, University of Catania, Viale Andrea Doria 6, 95125 Catania, Italy; 4 Regional Reference Laboratory of Clinical and Environmental Surveillance of Legionellosis, Azienda Ospedaliero Universitaria Policlinico “G. Rodolico-San Marco”, Via S. Sofia 78, 95123 Catania, Italy

**Keywords:** *Legionella*, biosensing technologies, environmental samples, systematic review

## Abstract

The detection of *Legionella* in environmental samples, such as water, is crucial for public health monitoring and outbreak prevention. Although effective, traditional detection methods, including culture-based techniques and polymerase chain reaction, have limitations such as long processing times, trained operators, and the need for specialized laboratory equipment. Biosensing technologies offer a promising alternative due to their rapid, sensitive, cost-effectiveness, and on-site detection capabilities. To summarize the current advancements in biosensor development for detecting *Legionella* in environmental samples, we used ‘*Legionella*’ AND ‘biosensors’ NEAR ‘environmental samples’ OR ‘water’ as keywords searching through the most relevant biomedical databases for research articles. After removing duplicates and inadequate articles from the n.1268 records identified using the PRISMA methodology exclusion criteria, we selected n.65 full-text articles which suited the inclusion criteria. Different results between the studies describing the current biosensing techniques, including optical, electrochemical, magnetic, and mass-sensitive sensors were observed. For each biosensing technique, sensitivity, specificity, and detection limits were evaluated. Furthermore, the integration of nanomaterials, microfluidics, and portable devices in biosensor systems’ design were discussed, highlighting their role in enhancing detection performance. The potential challenges and future directions in the field of *Legionella* biosensing were also addressed, providing insights into the feasibility of implementing these technologies in routine environmental monitoring. Undoubtedly, biosensors can play a crucial role in the early detection and management of *Legionella* infections and outbreaks, ultimately protecting public health and safety.

## 1. Introduction

*Legionella pneumophila* is an environmental pathogen that can lead to Legionnaires’ disease, a severe pneumonia associated with high mortality rates, which can occur in 41% for patients with an underlying condition [1]. *Legionella* is transmitted to humans through droplets produced from environmental sources such as showerheads, cooling towers, spas, whirlpools, and other aerosol-generating human-made devices [2]. Therefore, the detection of legionellae by water sampling is important in epidemiological investigations of Legionnaires’ disease and its prevention [3]. In addition, the epidemiological investigation is crucial in the risk evaluation for the implementation of a water safety plan (WSP) [4,5] and the choice of the best disinfection method [6,7,8].

Today, “the gold standard” for the identification of *L. pneumophila* is the culture method. Nevertheless, many drawbacks can be found using this methodology, like the presence of viable but non-culturable cells (VBNC), which are potentially pathogenic but cannot be detected with culture, the loss of viability of the bacterium after collection, difficulties in isolation because other bacteria grow faster disguising *Legionella* contamination, and the long time (5–15 days or more) required for culture and confirmation [9,10,11]. Other detection strategies can be used for *Legionella* monitoring, and among them, it is worth mentioning quantitative polymerase chain reaction (qPCR) [12,13], loop-mediated isothermal amplification (LAMP) [14,15], and more recent immuno-based assays (ELISA) [16], which provide results in few hours. However, despite their high sensitivity and specificity, these techniques have some drawbacks: they need sampling and expert operators, they are expensive and sometimes time-consuming, and they are not able to discriminate between dead and live cells [17,18,19].

There is an increasing necessity to develop portable and easy-to-use devices with high sensitiveness, specificity, and a rapid feedback time, to continuously monitor water systems [20,21]. To detect and monitor *L. pneumophila* in water samples, some features are needed for developing new detection systems: fast results, low cost, no necessity for experienced personnel, and a sensitivity similar to the culture method. The answer to all these needs could be represented by biosensors, which are analytical devices that can reveal biological compounds like cells, enzymes, proteins, and antibodies, as well as DNA, at low cost, and with high sensitivity and specificity. The ‘ideal’ biosensor should reveal low traces of a compound to be detected and should be able to discriminate among *Legionella* species and serogroups [22,23,24].

This systematic review points out the several types of biosensors that are now available for the fast detection of *Legionella* in water samples. Their characteristics, advantages, and areas for improvement are taken into consideration to have a clear and upgraded overview.

## 2. Materials and Methods

Following keywords have been chosen searching for literature: ‘*Legionella* and biosensors’ OR ‘biosensors’ NEAR ‘*Legionella* detection’ AND ‘water’ OR ‘environmental samples’ NEAR ‘*Legionella*’ AND ‘biosensors’ AND ‘biosensing’. The Preferred Reporting Items for Systematic Reviews and Meta-Analyses (PRISMA) guidelines were followed [25].

**Figure 1:**
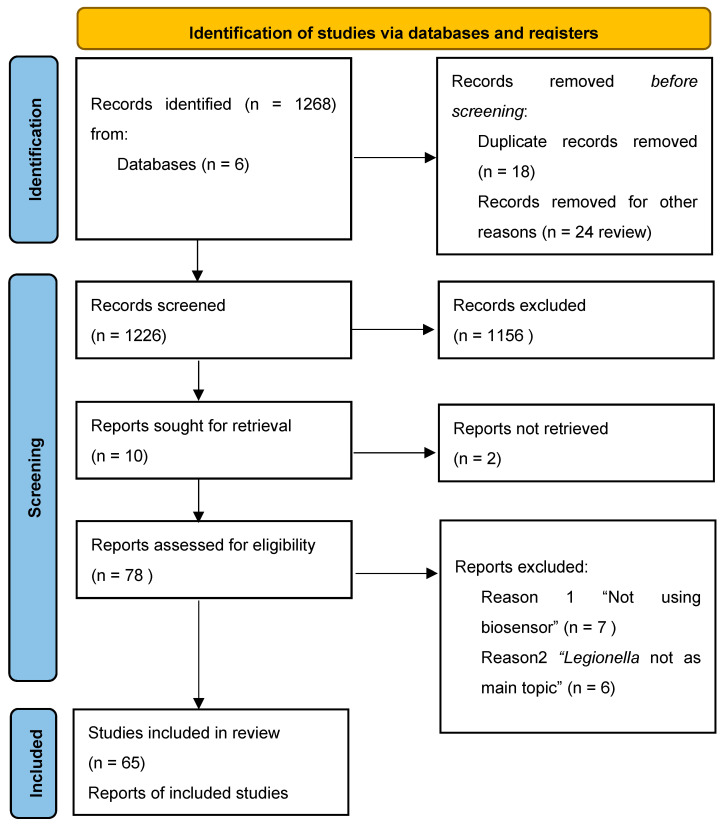
Study selection flowchart following the PRISMA guidelines.

The search included papers published between 1980 and the first months of 2024, and it was carried out in relevant biomedical databases: ACS Publications, Elsevier, JSTOR, PubMed, SDOS, and Wiley Online Library. The following inclusion criteria were adopted: (i) detection of *Legionella* in environmental samples by biosensors, (ii) indication of a limit of detection (LoD), (iii) indication of processing times, and (iv) description of operation of the biosensor. The non-eligibility criteria used were (i) review articles, (ii) non relevant results in terms of processing time and LoD, (iii) no indication of bias or limitation of the study, and (iv) funded studies. The literature review was classified into three main parts to assess the application of biosensors for the detection of *Legionella* in water: (i) type of biosensors, (ii) type of bioreceptor, and (iii) type of transducer. As shown in Figure 1, n.1268 records were selected for further review after the first search through databases. Once the duplicates (n.18) and the review articles (n.24) were removed, a total of n.1156 records were excluded because they did not match the topic. Eventually, after retrieving n.8 reports, among the n.78 full-text articles screened, n.65 were selected because they suit the inclusion criteria.

Finally, an additional n.36 articles were used to highlight the importance of the topic in the Introduction and to provide a general description of the individual methodologies. 

## 3. Biosensors as an Alternative Tool to Conventional Methods in the Detection of *Legionella*

In recent decades, a wide variety of biosensors have been developed for numerous applications, including environmental pollutant monitoring, the food industry, fermentation processes, saccharification, medical and pharmaceutical uses, metabolic engineering, and plant biology [26]. Significant efforts have also been directed towards creating biosensors for the rapid detection of *Legionella* in water samples. The broad adoption of these technologies is attributed to their superior sensitivity and specificity compared with traditional methods. Biosensors consist of two primary components: a bioreceptor or biorecognition element, which is specific to the target analyte such as proteins, enzymes, cells, antibodies, or DNA, and a transducer, which converts the recognition event into a measurable signal, typically electrical. Bioreceptors can vary in their biological nature, being composed of microorganisms, organelles, tissues, cells, antibodies, enzymes, or nucleic acids, while the transduction process may involve electrochemical, optical, piezoelectric, thermometric, magnetic, or micromechanical methods, or a combination of these techniques.

Biosensors can be classified depending on bioreceptors or transducers. 

### 3.1. Bioreceptor Categories

The bioreceptor is the key element for recognition and provides the sensitivity of the biosensor. The different types of bioreceptors can be grouped into five main categories: nucleic acids, enzymes, antibodies, whole cells, and biomimetic receptors.

- Nucleic acids

DNA and RNA are used as biorecognition elements thanks to the complementarity of the base pairs. Nucleicacid sequences are used because they link to specific genes that are characteristic of a peculiar species [27]. They can be detected using various transducers depending on the chemical-physical characteristic to detect. Examples include mass change (using quartz microbalance), optically if markers are linked to the target molecule, or electrochemically thanks to the charge variation due to the link.

- Enzymes

Enzymes link specifically to their substrate. They catalyze a reaction (usually an oxy reduction) to generate an electron transfer to be detected by a working electrode [28].

- Antibodies

Antibodies are used to link specific molecules (antigens). They can be monoclonal, polyclonal, or recombinant, based on their binding properties and the methodology used to synthetize them [29]. The transduction mechanisms mostly used are mass change, optically if markers are linked to the target molecule, or using surface plasmon resonance.

- Whole cell bioreceptors

Eukaryotic and procaryotic cells can be used as biorecognition elements to detect chemical pollutants, enzymes, and bacteria. Biorecognition occurs when a whole cell or a cellular component bind specifically to certain analytes [27].

- Biomimetic receptors

Biomimetic receptors are molecules that simulate enzymes, DNA, cells, and antibodies with similar affinities and specificities in the biorecognition process. They are highly selective towards the target molecules and, in principle, they can be synthesized for any analyte molecule to bind [30].

### 3.2. Types of Transducers

There are five main groups of transducers, which in turn can be divided into different subtypes (Table 1): optical, electrochemical, magnetic, and mass-sensitive measurement, depending on the transducing process.

## 4. Optical Biosensors

Optical biosensors are a sophisticated class of devices that utilize optical phenomena to detect and measure specific biological molecules or biochemical processes. These sensors are extensively used across various fields, including medical diagnostics and environmental monitoring, and are valued for their high sensitivity, specificity, and their capacity to provide rapid, user-friendly, portable, multiplexed, and cost-effective diagnostics. The working principle of optical biosensors is based on the interaction between light and the target biomolecules. These devices typically employ a biological probe, such as antibodies, enzymes, or nucleic acids, which is immobilized on a sensing surface. When the target biomolecule binds to the biological probe, this interaction results in several measurable optical changes, including shifts in reflectance, absorbance, or fluorescence. Optical biosensors can be classified into different types, including colorimetric, photoluminescence, chemiluminescence, fluorescence, MCDA-LFB, surface plasmon resonance (SPR), and photonics-based sensors.

### 4.1. Colorimetric

Colorimetric optical biosensors detect specific biological molecules or biochemical processes by using color changes as indicators. In these devices, a biological probe—such as an antibody, enzyme, or nucleic acid that can selectively bind to a target molecule like an antigen or substrate—is immobilized on a sensor substrate, which could be made of materials like glass, silicon, or other optically transparent substances. When the target molecule is present in the sample, it interacts with the immobilized biological probe on the sensor’s surface. This interaction forms complexes that cause measurable optical changes, which can influence the substrate’s reflectance, absorbance, or fluorescence, resulting in a visible color change. The resulting color variation can be observed directly or measured with tools such as a spectrophotometer or camera. In some cases, the intensity or hue of the color change correlates with the concentration of the analyte, allowing for the determination of its presence and quantity based on a color scale or data obtained from the measurements. Additionally, comparisons with known control samples can be used to enhance the accuracy of the results.

In this review, we compared the results obtained in terms of the detection limit from three studies that exploited the colorimetric technique to detect *L. pneumophila* with an optical biosensor. Bedrina et al. (2013) [30] employed a technique combining magnetic immunocapture and enzyme-immunoassay for rapid *L. pneumophila* detection in water samples. Magnetic microspheres coated with anti-*L. pneumophila* antibodies underwent filtration, resuspension, immunomagnetic capture using immune-activated beads, and colorimetric enzyme-linked immunodetection in just 1 h of analysis. The results demonstrated 96.6% sensitivity, 100% specificity, 0% false positives, 3.4% false negatives, and an efficiency of 97.8%. The detection limit was established at 93 colony-forming units (CFU) in the examined volume. This method proved to be a rapid screening technique, maintaining a performance comparable to conventional culture for *L. pneumophila* isolation. Years after, Nuthong et al. (2018) [31] used a colorimetric approach utilizing gold nanoparticles (AuNPs) conjugated with thiolated DNA probes for rapid *L. pneumophila* detection. Mixing *L. pneumophila* with the probes, the result was a red solution easily detectable by the naked eye, with color stability upon the addition of MgSO_4_. The results demonstrated a negative predictive value of 100%, a sensitivity of 100%, and a detection limit of 45 ng DNA μL^−1^ with a rapid, 6 min response time for 124 suspected *Legionella* colonies. Finally, a membrane filter method was developed by Párraga-Niño et al. (2018) [32] for capturing *L. pneumophila* in water samples. In this system, the bacteria were retained using a nitrocellulose disc inside a homemade cartridge and then immunodetected. 3,3′,5,5′-Tetramethylbenzidine, a colorimetric substrate, was added to the membranes and incubated in the dark for 16 min. Finally, using an ELISA reader that measured absorbance at 450 nm to quantify the substrate reaction, great specificity and sensitivity for all *L. pneumophila* serogroups was reached. In particular, a monoclonal antibody detected an LoD of 350 CFU/L.

### 4.2. Photoluminescence

Optical photoluminescent biosensors are devices that utilize the light-emitting properties of certain materials, such as quantum dots, fluorescent molecules, or metallic nanoparticles, when they are excited by an energy source like ultraviolet (UV) light. In these biosensors, a biological probe, which is specific to the target molecule (such as proteins, metal ions, enzymes, DNA, or RNA), is immobilized on the surface of the photoluminescent material. When the target molecule interacts with the probe, it can alter the photoluminescence of the material. To initiate this process, the biosensor is exposed to an energy source, commonly an ultraviolet LED or a laser, which causes the photoluminescent material to emit light. The emitted light is then detected and measured. The interaction between the target molecule and the probe affects the material’s photoluminescence, generating a signal that can be quantified. This signal is correlated with the concentration of the target molecule in the sample, allowing for the determination of its presence and concentration.

Islam et al. (2022) [33] explored an innovative biosensing method utilizing digital photo-corrosion (DIP) of GaAs/AlGaAs semiconductor nanoheterostructures for the detection of *L. pneumophila*. The method relies on the sensitivity to the charge transfer between the immobilized biomolecules and a semiconductor. By coating *L. pneumophila* with negatively charged sodium dodecyl sulfate (SDS), and using polyclonal antibody (pAb)-functionalized DIP biochips, the detection was achieved at 10^3^ CFU/mL. The bacteria-coated biochips were exposed to a 660 nm LED. A measurement of the photoluminescence of intermittently irradiated biochips for 5 s irradiation in a 20 s total period was conducted for photo corrosion monitoring using an intensity-homogenized beam that transfers a power density of ~17 mW/cm^2^ to the biochip surface.

Islam et al. (2021) [34] manufactured a biosensor using a cysteine-modified Warnericin RK antimicrobial peptide (Cys-AMP) for the detection of *L. pneumophila*. The biosensing architecture involved functionalizing GaAs/AlGaAs nanoheterostructure biosensors based on digital photocorrosion. FTIR (Fourier-transform infrared spectroscopy), AFM (Atomic Force Microscopy), XPS (X-ray Photoelectron Spectroscopy), and water contact angle measurements were conducted to investigate the impact of peptide concentration on the ability of capturing *L. pneumophila*. Using optical microscopy, it was shown that 50 μg/mL of Cys-AMP was the optimal concentration for the maximum capture of *L. pneumophila.* The biosensor’s detection sensitivity was explored between 10^2^ and 10^6^ CFU/mL of *L. pneumophila*, estimating LoDof 2 × 10^2^ CFU/mL. Azyzian et al. (2016) [35] also efficiency employed GaAs/AlGaAs nanoheterostructure biosensors for detecting *L. pneumophila.* Since charged functional groups are essential for charge-sensing biosensors determining the net electric charge of the bacteria, the authors used negatively charged sodium dodecyl sulphate (SDS) molecule binding to bacteria to study the dynamic range of bacterial electric charge variations. A measurement of the doubled zero potential of *L. pneumophila* at pH 7.4 was conducted, exposing the bacteria to an SDS solution at 0.02 mg/mL. This led to the detection of *L. pneumophila* with an LoD of 10^3^ CFU/mL.

### 4.3. Chemiluminescence

Optical chemiluminescence biosensors utilize the chemiluminescence process to detect specific biological molecules or biochemical activities. Chemiluminescence occurs when light is emitted as a result of a chemical reaction. These biosensors combine biological recognition with light generation to produce a detection signal. A biological probe, specific to the target molecule, is immobilized on the surface of a substrate made of optically transparent materials like glass or silicon. When the target molecule is present in the sample, it interacts with the immobilized probe on the biosensor’s surface, triggering a chemical reaction that produces a substance in an excited state. As this substance returns to its ground state, it releases energy in the form of light, creating chemiluminescence. The light emitted from this chemiluminescence reaction is then detected by an optical system, such as a camera or photodetector. The intensity of the light emission is proportional to the amount of the target molecule in the sample, allowing for the quantification of its concentration.

Yoon et al. (2003) [36] developed a photometric immunosensor for on-site measurements by assembling a sensor system with a nitrocellulose membrane strip (bottom part) containing an immobilized antigen, and a glass fiber membrane strip (top part) with two electrodes. To enhance the signal, a liposome with ruthenium molecules and an antibody specific to a recombinant lipoprotein of *Legionella* was introduced. Upon the presence of the analyte, immune complexes took place through antibody/antigen binding. Capillary action absorbed this mixture from the membrane strip’s bottom, carrying liposome particles through the antigen pad without interaction, while free immuno-liposomes were stuck on the pad surfaces. Apre-located detergent was dissolved by the influx of the aqueous medium into the glass pad, causing liposome rupture and releasing ruthenium molecules into the solution. Oxidation on the electrode surfaces produced an electrochemiluminescence reaction (ECL) proportional to the analyte concentration. The ECL-based signal generation exhibited an exponential dose-response pattern, with a detection limit of 2 ng/mL, with a sensitivity about 10 times higher than a colorimetric system. Finally, a chemiluminescent DNA microarray capable of quantifying viable and non-viable *Legionella* spp., as well as *L. pneumophila* within 1 h, was developed by Kober et al. (2018) [37]. Heterogeneous asymmetric recombinase polymerase amplification (haRPA) was performed on these chemiluminescent DNA microarrays and detection limits of 87 genomic units (GU)/μL and 26 GU/μL for *Legionella* spp. and *L. pneumophila* were reached, respectively.

### 4.4. Fluorescent Optical Biosensors

Fluorescent optical biosensors are devices utilizing materials or molecules emitting fluorescence when excited by an energy source, such as ultraviolet or visible light. An optical system, such as a camera or a photodetector, measures the intensity of the emitted light, which is proportional to the target concentration. These biosensors typically exhibit higher sensitivity than most colorimetric biosensors due to the specificity of the emitted light and the presence of external instrumentation to reveal it. In recent times, nanomaterials, like, for example, gold nanoparticles, silver nanoparticles, quantum dots, or carbon dots, have found application in optical biosensors. These nanomaterials exhibit outstanding chemical, physical, and optical characteristics, rendering them well-suited for achieving low detection limits and high sensitivity, contingent upon their fluorescence conversion properties. 

Martynenko et al. (2019) [38] used quantum dots combined with superparamagnetic iron oxide nanoparticles and mesoporous calcium carbonate microbeads surface-functionalized with specific antibodies for *L. pneumophila.* This system showed great specificity towards *L. pneumophila* with an LoD lower than 100 CFU/mL and only very weak unspecific cross-reactivity with *E. coli.*

Wu et al. (2016) [39] measured DNA samples through a modified hybridization method, eliminating the need for prior DNA amplification. The system employed two target probes, conjugated with quantum dots having different emission spectra. These probes exhibited high specificity and sensitivity, with a detection limit of 10 CFU/test of *Legionella* spp.

A successful multiplex RNA assay was conducted by Foudeh et al. (2015) [40] utilizing digital microfluidics to specifically detect *Legionella* spp. by targeting 16S rRNA. The assay involved simultaneously manipulating multiple droplets on a chip. For each target RNA sequence, a fluorescently tagged DNA probe was designed. The fluorescence intensity of the droplets within the chip was measured using an inverted fluorescence microscope. All images were captured and analyzed, with measurements focused on the target droplet under the microscope showing a limit of detection of 1.8 aM of synthetic 16s rRNA and being able to distinguish among different species of *Legionella*. 

Rothenbroker et al. (2021) [41] discovered a fluorogenic DNAzyme, identified as LP1, which exhibited reactivity towards numerous strains of *L. pneumophila*, while not exhibiting it against 25 other prevalent bacterial species, with a detectable signal in as few as 10 CFU/mL of *L. pneumophila*. Eisenreichova et al. (2023) [42] utilized dual-color fluorescence cross-correlation spectroscopy to examine the binding properties of recombinant fluorescent biosensors, LactC2 and P4M, with liposomes of different compositions. The focus of the study was SidM, a protein secreted by *L. pneumophila*, which utilizes phosphatidylinositol-4 phosphate to bind itself to the *Legionella*-containing replicative vacuole in host cells. Islam et al. (2020) [43] employed fluorescence in conjunction with antimicrobial peptides to directly detect on-site *L. pneumophila* in water samples, using GaAs/AlGaAs biochips. The peptides were covalently immobilized on the monolayer-functionalized GaAs surface. Fluorescence microscopy and a digital photocorrosion GaAs/AlGaAs biosensor were employed to investigate the efficiency of the specific interaction between the peptide and *L. pneumophila*. The proposed biosensor architecture enabled detection levels between 10^3^ and 10^6^ CFU/mL. Epifluorescence played a central role in an innovative method for detecting viable *L. pneumophila* and other *Legionella* spp. in water. This method developed by Delgado-Viscogliosi (2005) [44] combined a bacterial viability test with specific staining using antibodies. Different wavelengths were emitted: green for the viability stain and red for the specific stain. This immunological double staining method was able to distinguish among various *Legionella* spp. with an LoD of <176 *Legionella* cells/L.

*Legionella* bacteria display distinct fluorescence patterns for each species when exposed to UV excitation light. Honda et al. (2022) [45] developed a bacterial sensing system utilizing a fluorescence sensor to identify *Legionella* in water samples. The system included a photogate-type optical sensor equipped with a photocurrent measurement system, a circuit board with a current-voltage and an analog-to-digital converter, and a microcomputer. Depending on the wavelength of the LED light source, the current ratio generated in the sensor changed, demonstrating that the system works as a spectrometer able to distinguish between *L. dumoffii* and *L. erythra*. Yamaguchi et al. (2017) [46] integrated a microfluidic system for both on-chip fluorescent staining and the semi-automated detection of *L. pneumophila*. They implemented a portable system for cooling-tower waters’ on-site monitoring. The selective detection of *L. pneumophila* sg1 was achieved using a fluorescently labeled polyclonal antibody. The system’s detection limit was 10^4^ cells/mL but through water filtration of 0.5–3 L of the water sample, it was possible to obtain concentrations of *L. pneumophila* cells between 10^1^ and 10^3^ cells/mL.

Alhogail et al. (2021) [47] utilized proteases from the culture supernatants of *Legionella* spp. as biomarkers, supplementing them with specific fluorogenic peptide substrates. The system showed a relevant raise in fluorescence intensity upon proteases cleaving the fluorogenic substrates. The conjugation of these substrates with nano-magnetic particles (NMPs) on a gold surface was then performed. When exposed to the proteases, the peptide sequence undergoes digestion, causing the NMPs to displace from the gold surface, resulting in a gold color. With this system, great sensitivity was reached detecting as low as 60 CFU/mL of *L. anisa*, *L. micdadei*, *L. dumoffii*, and *L. pneumophila*.

Chawich et al. (2022) [48] assessed the biofunctionalization of GaAs/AlGaAs nano-heterostructures with polymer brushes as a platform to enhance the detection of *L. pneumophila* using a DIP biosensor, constructed by depositing metallic nanoparticles onto the tip of a cleaved optical fiber. The authors reported the successful detection of *L. pneumophila* at 500 CFU/mL. Curtin et al. (2023) [49] applied fluorescence techniques for biosensing in conjunction with Clustered Regularly Interspaced Short Palindromic Repeats (CRISPR)-associated effectors, such as Cas12a. In CRISPR-Cas biosensing, nucleic acid targets are identified by Cas nucleases through complementary binding with CRISPR guide RNA. Once binding occurs, the nuclease is activated, initiating collateral cleavage activity that cuts non-targeting DNA. Subsequently, single-stranded fluorophore-quencher DNA reporters are introduced to produce a clear fluorescence signal when Cas cleaving collateral activity occurs. This method was tested using not only *Legionella* but also Shigella, Campylobacter, and *Vibrio cholerae*, showing high specificity and an LoD for *Legionella* of 1 fg/mL. Daneshvar et al. (1999) [50] developed a method employing a fluorescent fiber-optic immunosensor (FFOI) for detecting antigen–antibody interactions in the near-infrared spectral region. In this approach, the FFOI’s sensing tip immobilizes a rabbit anti-LPS1 antibody, serving as a recognition element for trace amounts of a *Legionella* specific antigen. A semiconductor laser excited the immune complex, and a silicon photodiode detector captured the resulting emission. The fluorescence intensity correlated directly with the *Legionella* antigen concentration with an LoD down to 0.5 ng/mL.

Lee et al. (2022) [51] introduced a biological sensor named “tpMetROG”, designed to quantify oxidized methionine in the form of methionine-R-sulfoxide within target proteins. The study focused on the analysis of methionine-rich proteins from *L. pneumophila* (LegP), the lowest detected concentration of which was 1 μM. Finally, flow cytometry and fluorescence microscopy were utilized to validate the binding specificity of aptamers to *L. pneumophila*. Aptamers, short sequences of oligonucleotides that fold into specific structures, possess the ability to selectively attach to particular molecules. A study conducted by Saad et al. (2020) [52] employed Systemic Evolution of Ligands through EXponential enrichment (SELEX) to discover aptamers with specific binding to *L. pneumophila*. Two aptamers demonstrating strong binding to *L. pneumophila* were observed, exhibiting dissociation constants (KD) of 116 and 135 nM.

### 4.5. MCDA-LFB Assay

An innovative optical test was developed by Jiang et al. (2022) [53] coupling two techniques to detect the *mip* gene of *L. pneumophila* strains, indicated as MCDA-LFB. It consists of Multiple Cross-Displacement Amplification (MCDA), the anisothermal nucleic acid amplification technique, and a nanoparticle-based Lateral Flow Biosensor (LFB) technique. The entire process takes less than 1 h, consisting of 20 min of DNA preparation, 35 min of *L. pneumophila*-MCDA reaction, and 2 min of sensor strip reaction. For the test, 88 specimens of sputum, alveolar lavage fluid (from hospitalized patients), and water samples were used. The results were confirmed by comparing them with those obtained from *L. pneumophila* and non-*L. pneumophila* strain-infected samples from the laboratory. MCDA allowed the amplification *mip* gene using 10 specific primers, with an optimal amplification temperature of 65 °C monitoring the reaction with the real-time turbidity test. The presence of *Legionella* DNA was confirmed using LFB assay. The presence of only one crimson line, which stands for the control line, showed that the amplification reaction correctly occurred, while two lines indicated the positive result of the test because target analytes let reporter molecules aggregate on the test zone, causing a line on the test zone to appear. The developed strategy in the reported study detected five (5.68%) positive samples with LoDs of 10 fg of DNA, which completely covered the four positive samples detected by PCR and results were completely consistent with the conventional culture method. In addition, no cross-reaction occurred in the test.

### 4.6. Surface Plasmon Resonance (SPR)

Surface plasmon resonance (SPR) transducers have been widely used over the las few years. The optical characteristics of plasmonic biosensors largely depend on the surfaces of the substrates employed. An SPR-based biosensor can measure the disruption of coupled incident light to surface plasmons caused by the presence of compounds on metal−dielectric interfaces. Generally, SPR sensors have low sensitivity due to the limited penetration distance of the wave created at the interface into the volume of the sample. Moreover, refractive indices of bacteria and their medium are often similar, resulting in low sensitivity. Various methods have been developed to increase the LoD in SPR-based bacteria detection. Using this method, Oh et al. (2003, 2005) [54,55] developed both an immunosensor and a protein chip. The fabrication of self-assembled protein G layer on gold (Au) substrate was performed to direct antibody molecules on SPR sensor surfaces. Protein G, a cell wall protein present in many species of Streptococci, works as the antibody-binding protein. SPR spectroscopy was used to confirm the formation of the self-assembled protein G layer on a Au substrate, and the interaction between antibody and antigen. An atomic force microscope (AFM) was employed to analyze the surface morphology of the self-assembled protein G layer on a Au substrate and of a monoclonal antibody (Mab) against *L. pneumophila* immobilized on protein G. The limit of detection was 10^5^ cells/mL. Lin et al. (2007) [56] developed an immunosensor that uses side-polished optical fiber based on SPR to detect L. pneumophila using an 850 nm LED with a detection limit of 101 CFU/mL. A SPR prototype provided with miniaturized biochips and microfluidic circuits for assessing the on-site monitoring of water samples was developed by De Lorenzis et al. (2013) [57]. After an accurate characterization of the presented immunoassay by ELISA, realization of the SPR prototype instrumentation occurred. The study shows that the integration of microfluidic technologies with SPR-based biosensing setups is possible, resulting in a cost-effective and portable biosensor candidate. The manufactured immunosensor was able to sensitively detect L. pneumophila in aqueous solutions, with a limit of detection of 10^3^ cells/mL. Filion-Côté et al. (2014) [58] showed a SPR biosensor operating in the visible spectrum. The authors presented preliminary tests to demonstrate that the device works efficiently and that it accurately reveals changes in the refractive index in real time. The simulations predicted that a system that operates in the visible spectrum with a higher bit depth and a higher spatial resolution would have a better efficiency than a device working in the infrared. The experimental detection limit of the SPR biosensor presented was 9.8 × 10^−7^ refractive index unit (RIU). 

More than one study has used surface plasmon resonance imaging (SPRi) instead of classical SPR. This method allows for the visualization of many interactions simultaneously in real time due to the integration of a charge-coupled device (CCD) camera with the associated sensogram. In particular, 16s rRNA was used for the identification of *L. pneumophila*, because it is a good representation of metabolically active bacteria. Foudeh et al. (2014) [59] showed that sub-femtomole levels of 16s rRNA from pathogenic *L. pneumophila* can be precisely revealed using an accurate protocol, an adequate design of the capture and detector probes, and using a quantum dot (QD) signal amplification system with an SPRi biosensor. There are at least three main advantages in using these detections strategies compared with standards methods: great specificity towards the design of two probes for the target, raised sensitivity through using QD signal post amplification, and fast and reliable quantification employing *L. pneumophila* 16s rRNA. The LoD of the 16s rRNA was 1 pM, which is equal to 88.5 CFU/µL. In another study, Foudeh et al. (2015) [60] reported the detection of synthetic 16s rRNA from *Legionella* employing digital microfluidic (DMF) devices. The authors showed that the developed assay could achieve great selectivity by illustrating the amplification-free and multiplex detection of rRNA from two different species of *Legionella*, and in particular *L. pneumophila* and *L. israelensis*. The study showed the outstanding connection of the RNA assay in DMF for the specific detection of *Legionella* spp. using 16s rRNA targets. This combination demonstrated a great potential for the fast, multiplex, high-throughput, and low-cost detection of pathogenic microorganisms with low sample and reagents amounts. The limit of detection of 16s rRNA was 1.8 attomoles. Melaine et al. (2017) [61] developed a novel biosensor for *L. pneumophila* using SPRi in which gold nanoparticles (GNPs) and a DNA probe sandwich were assembled to amplify transduction signals. 16S rRNA of *L. pneumophila*, *Pseudomonas aeruginosa*, and *Salmonella typhimurium* were used as the target of GNP-grafted DNA detection probes. This technique proved the implementation of 16S rRNA detection strategies with high specificity and an LoD down to 10 pg/mL. A new sensitive strategy for detecting *L. pneumophila* was presented by Meneghello et al. (2017) [62]. In the study presented, azimuthally controlled grating-coupling SPR technology (GC-SPR) was optimized and employed to detect *L. pneumophila* utilizing sinusoidal gold gratings. Sensitivity was 1000 times higher than the one reached using fluorescence techniques. In particular, the GC-SPR proved its great ability in detecting down to 10 CFU. Negative control experiments with *Escherichia coli* also demonstrated the high specificity of the proposed biosensor. Karimiravesh et al. (2022) [63] developed an optical nano biosensor in which thiolated probes created for the *mip* gene of *L. pneumophila* were linked to gold nanoparticles and then water samples with bacteria were monitored. The obtained detection limit of DNA was 10^3^ copy number/µL. Another experimental technique was developed by You et al. (2020) [64] to increase the localized surface plasmon resonance (LSPR) color change. The authors examined the effect of size and number density of aggregating AuNPs on enhancing the LSPR color-change signal and feedback time and sensitivity of an immuno-biosensor. The efficiency of this test was proved by detecting streptavidin, a model protein, and two bacteria, *E. coli* and *L. pneumophila*, with an LoD of 10 CFU/mL for both bacteria.

### 4.7. Photonic Technology

Photonic crystals (PCs) have been extensively used for optical sensors but some PC-based platforms involve sophisticated techniques, hazardous chemicals, and high-cost apparatus. To overcome these drawbacks, ‘printable photonics technology’, consisting of nano-imprint lithography (NIL), was presented to fabricate optical nanodevices. NIL is a cheap and high-performance technology used to fabricate nanometer-scale patterns by employing polymers with high homogeneity. Li et al. (2013) [65] investigated the use of NIL-based PC surface immunochip fabrication, which are able to detect *L. pneumophila*. The authors produced two-dimensional PCs on a copolymer film connected with Fresnel reflection spectroscopy and fluorescence microscopy. By employing a PC platform, a detection limit of 200 *L. pneumophila* cells/mL was reached.

## 5. Electrochemical Bacterial Biosensors

Electrochemical biosensors detecting bacteria analyze interactions between bioreceptors and bacteria investigating electrical signals changes (e.g., potential, current, impedance, and capacitance). These biosensors are made of three electrodes: a reference, a counter, and a working electrode. The working electrode is the most important part of the electrochemical biosensor; it can be produced from the nanometer to millimeter scale as well as from various materials (e.g., carbon and gold) and can also be functionalized to increase specificity and sensitivity [95].

### 5.1. Amperometric Biosensors

Amperometric biosensors are integrated tools that operate autonomously and are able to measure the current generated from the reduction or oxidation of an electroactive biological element supplying precise quantitative information. Charge transfer can occur directly from target molecules or by indirect signaling through redox reactions catalyzed by enzyme labels on the target analyte [96].

A novel, easy-to-use, sensitive, and in-a-day electrochemical system to detect *L. pneumophila* sg1 in water was developed by Martin et al. (2015) [66]. The authors described an innovative amperometric-magneto-immunoassay which combined the use of poly(dopamine)-modified magnetic nanoparticles (MNPs@pDA) and screen-printed carbon electrodes (SPCEs). A specific capture antibody (Ab) was linked to the MNPs@pDA followed by incubation with bacteria. The captured bacteria were sandwiched using the antibody labeled with horseradish peroxidase (Ab-HRP). Capture of the resulting MNPs@pDA-Ab-*Legionella pneumophila*-Ab-HRP took place after a magnetic field was applied on the surface of the electrode. The achieved LoD was 10 CFU/mL.

In the field of amperometric biosensors, other techniques improve bacterial detection, like the thioaromatic-based oligonucleotide monolayers on gold, which propose a convenient methodology to directly detect rRNA with no need for preliminary amplification. Miranda-Castro et al. (2017) [67] investigated different approaches involving self-assembled monolayers (SAMs) of aromatic thiols, specifically p-aminothiophenol (p-ATP) and p-mercaptobenzoic acid (p-MBA), to produce DNA-sensing layers to detect target oligonucleotides. The proposed monolayers were assessed by DNA surface coverage and their functionality was evaluated in a sandwich hybridization assay with an enzymatic amplification of the electrochemical read-out. Thanks to the use of the covalent binding of amino-oligonucleotides to pure p-MBA monolayers and thiol-oligonucleotides into p-ATP monolayers previously oxidized, improved storage stability and analytical performance occurred. RNA quantification coming from *L. pneumophila* cellular lysates was successfully carried out, showing the threat potential of these sensing systems for pathogenic bacteria detection. Ezenarro et al. (2020) [68] showed the application of an electrochemical system consisting of a mixing of sample concentration, immunoassay detection, and chronoamperometry measurement. Specifically, a nitrocellulose microfiltration membrane was employed for the support of the sample concentration and the antigen–antibody reaction. Labelling of anti-*Legionella* antibody for the biorecognition was performed using the horseradish peroxidase (HRP) enzyme, with the help of a mediator (3,3′,5,5′-Tetramethylbenzidine—TMB), thus observing current changes related to the concentration of *Legionella* in the samples. The detection limit was 4 CFU/mL. Another work by Mobed et al. (2019) [69] led to the fabrication of a novel genosensor for detecting *Legionella*. Silver nanoparticle-graphene quantum dots (Ag/GQDs), nano-ink grafted by gold nanoparticles supported by Cysteamine A (CysA/AuNPs), towards the detection of the *mip* gene from *Legionella* was manufactured. Chronoamperometry was applied to monitor the hybridization of cDNA at different concentrations with the highest peak current at 50 µA for 1 µM of cDNA and the minimum one at 20 µA for a concentration of 1 zM, which is the low limit of quantification (LLOQ). Ag/GQDs nano-ink modified by CysA-AuNPs raised the number of immobilized probes (ssDNA) and increased the sensitivity of the genosensor for detecting dsDNA, while also discriminating the presence of mismatch in the target sequence.

### 5.2. Voltammetric Biosensors

Voltammetric methodologies are electrochemical methods in which current measurement happens after an application of electrical potential that directs redox reactions [97,98,99]. Different techniques have been conceived so far and, among them, the most frequently used in biosensing are cyclic voltammetry (CV), square wave voltammetry (SWV), and different pulse voltammetry (DPV). 

An ultrasensitive immunodevice based on a ZnO nanorod electrode was produced to detect *L. pneumophila* by Park et al. (2014) [70]. An evaluation by CV demonstrated that the fabricated electrochemical immunosensor can enhance the sensitivity monitoring of *L. pneumophila* with an LoD of ∼1 pg/mL. Olabarria et al. (2020) [71] developed a genosensor that combines a loop-mediated isothermal amplification (LAMP) reaction with an electrochemical transducer. The study proposed the selection of a specific genetic marker for *Legionella* spp. while electrochemical detection was performed using CV scans. This sensor allowed the detection of 10 fg of *Legionella* nucleic acid, corresponding to only two copy numbers of the bacteria.

Laribi et al. (2020) [72] experimented with an innovative microfluidic assay to continuously detect *L. pneumophila* sg1 in artificial water samples. A monolayer of 16-amino-1-hexadecanethiol was assembled and then linked to a gold substrate. This surface was used to anchor anti-*L. pneumophila* monoclonal antibodies. Characterization steps were performed using SWV, CV, and microscopic imaging methods and the biosensor showed great sensitivity in the range between 10 and 10^3^ CFU/mL under dynamic conditions.

Rai et al. (2012) [73] developed a DNA-based biosensor using alumina-modified electrodes. Electrochemical responses were analyzed using CV, showing an ultrasensitive detection of 21-mer complementary sequence (3.1 × 10^−13^ M) and the ability to discriminate between different serogroups of *L. pneumophila*. In another study, Rai et al. (2012) [74] identified an LoD of 2.3 × 10^−14^ M for the detection of a 21-mer DNA sequence, showing an increased sensibility compared with the previous study. In this case, the characterization process was carried out using DPV.

Miranda-Castro et al. (2009) [75] developed a PCR-coupled electrochemical sensor for *L. pneumophila*. Data obtained from this study showed that the voltammetric signal was related to the numbers of amplicons, achieving a semi-quantitative estimation of *L. pneumophila*. After 40 cycles, the sensor was able to detect up to 10 genomic units (GU), with reliable detection of 10^2^ GU. In a previous study, Miranda-Castro et al. (2008) [76] presented a voltammetric sensor to detect DNA sequences specific for *L. pneumophila.* CV was used for the characterization of the biosensor and a detection limit of 9.6 × 10^9^ copies of a 52-mer DNA sequence specific of *L. pneumophila* was proved.

Mobed et al. (2019) [77] fabricated a biosensor in which gold nanoparticles in the presence of cetyltrimethylammonium bromide and chitosan were electrodeposited on the surface of a gold electrode using the CV technique. SWV was used for the characterization using cDNA at different concentrations, showing great selectivity sensitivity and fast feedback time in *L. pneumophila* detection. The linear range of detection was 1 μM to 1 fM.

Other studies have focused on the fabrication of biosensors that use the DNA immobilization and hybridization technique combined with voltammetric methodologies. Mobed et al. (2019) [78] developed an innovative nucleic acid biosensor for the detection of *Legionella* using the *mip* gene as a target. The deposition of gold nanoparticles supported by cysteamine was performed on the surface of a gold electrode providing a wide area for the immobilization of ssDNA. CV and SWV were employed to electrochemically characterize DNA immobilization and hybridization by cDNA, revealing great sensitivity at a linear range from 1 μM to 1 zM. In another study, Mobed et al. (2019) [79] fabricated a new electrochemical DNA-based biosensor for *L. pneumophila* using gold nanoparticles and employing a transducer substrate connected with toluidine blue as a redox marker. DNA hybridization with cDNA was evaluated using SWV and DPV. Also in this case, the linear range of detection was from 1 μM to 1 zM.

In the last few years, carbon nanotubes (CNTs) have raised attention as electrode materials for voltammetry measurements with an enormous potential for high-sensitivity electrochemical biosensors thanks to their peculiar features, such as the promotion of electron-transfer reactions of biomolecules, great electrocatalytic activity, and reduced surface fouling effects. Park et al. (2010) [80] functionalized a multi-walled carbon nanotube electrode with oxygen plasma treatment and characterized it with CV for the detection of *L. pneumophila*. The DNA-based sensor was successful at detecting mip genes, ranging from 10 pM to 100 nM. Lee et al. (2010) [81] fabricated an electrochemical immunosensor-based working electrode using patterned plasma-functionalized multi walled carbon nanotubes (pf-MWCNTs) to detect *L. pneumophila*. Passive adsorption and covalent immobilization were used for polyclonal antibody adhesion and various dilutions of *Legionella* 19-kDa peptidoglycan-associated lipoprotein were tested for measurements. CV versus a silver/silver chloride (Ag/AgCl) reference electrode was conducted for electrochemical detection, resulting in 0.16 ng ml^−1^ and ∼0.0025 ng ml^−1^ LoD for passive adsorption and covalent immobilization, respectively. Subsequently, Lee et al. (2012) [82] developed a plasma-activated carbon nanotube for monitoring *L. pneumophila* through the detection of a maltose-binding protein peptidoglycan-associated lipoprotein (MBP-PAL). According to SWV, the electrochemical sensitivity of the immunodevice to MBP-PAL was 55.7% higher compared with PAL with an LoD of 5 pgmL^−1^. Finally, Kim et al. (2012) [83] used a single-walled carbon nanotube (SWCNT) three-electrode system as an electrochemical biosensor. The efficiency of miniaturized SWCNT electrodes was performed by using them for *L. pneumophila* detection based on a direct sandwich ELISA format. Electrochemical properties were performed using CV and chronoamperometry showing a detection limit of the sensor lower than 10 pgmL^−1^.

### 5.3. Impedance-Based Biosensors

Impedance is defined as the resistance an electrical circuit can carry out to a component in response to an alternating current. Impedance-based systems represent some of the most accurate and reproducible characterization methodologies for electrochemistry. Impedance measurements are applied by small voltage perturbations revealing the related current response. We refer to impedance as the ratio of the voltage-time function and the current-time function. The latter is evaluated using electrochemical spectroscopy (EIS), in which a variation in voltage frequency occurred [100]. So far, many impedance-based biosensors have been developed targeting several analytes. Lei et al. (2012) [84] designed an electrochemical sensor using a gold electrode with anti-*Legionella* sg5 IgG immobilized on its surface, demonstrating the detection of *Legionella* concentrations from 10^5^ to 10^8^ CFU/mL. 

Souiri et al. (2014) [85] employed monoclonal anti-*L. pneumophila* antibodies on an indium–tin oxide (ITO) electrode surface to create an electrochemical immunodevice to detect *L.pneumophila*. Epoxysilane-antibodies complexes were used as barriers for the electron between the electrode surface, increasing the charge transfer resistance (Rct), with an LoD of 5 × 10^1^ CFU mL. Sboui et al. (2015) [86] presented an immunosensor for *L. pneumophila* fabricated by the immobilization of a monoclonal anti-*L. pneumophila* antibody (MAb) on an ITO electrode by the self-assembled monolayers (SAMs) methodology utilizing an aminosilane. The characterization of the immunosensor was conducted using several techniques, and with the electrochemical impedance spectroscopy (EIS) technique, the authors reached an LoD of 10 bacteria/mL in artificial samples. Other studies were conducted using antibody-immobilized electrodes. Muhsin et al. (2022) [87] tested a biosensor for the detection of *Salmonella*, *Legionella*, and *E. coli O157:H7* in wastewater and tap water. Once the interaction of the bacteria to their specific antibodies occurred, changes in impedance value took place reaching a detection limit of 3 bacterial cells/mL. Li et al. (2012) [88] developed an immunochip system to monitor all *Legionella* serogroups in clinical and environmental samples. Covalent immobilization of fluorophore-conjugated *L. pneumophila* antibodies on Au chips was performed for biosensor preparation while detection was performed using impedance spectroscopy and fluorescence microscopy with a limit of detection of 2.0 × 10^2^ cells/mL. Su et al. (2018) [89] employed collagen-like (Lcl) protein, which is able to bind to extracellular matrix components and permits the binding of *L. pneumophila* to host cells, for developing a new sensor in which EIS/SPR-based biosensors were used to characterize the interactions between the adhesin Lcl protein and glycosaminoglycans (GAGs). Finally, an electrochemical DNA sensor was developed by Li et al. (2017) [90] for the contemporary detection of *Legionella* spp. and *L. pneumophila*. The immobilization of the hairpin probes took place by Au-S chemical bond reaction on the surface of AuE electrodes. These ones could be cleaved by EcoRI when *Legionella* was absent but not in the presence of *Legionella* spp. or *L. pneumophila*. The current changes depending on hybridization and enzymatic cleavage were monitored by the proposed DNA sensor distinguishing among the different *Legionella* species, with a limit of detection of 2 × 10^−9^ mol/L.

## 6. Magnetic

Magnetic-based detection is an effective method for biochemical sensing based on the magnetic field created by magnetic nanoparticles (MNPs), mainly iron oxide nanoparticles, that bind to target molecules in a biological assay. Usually, MPs are divided into ferromagnetic, paramagnetic, ferrimagnetic, anti-ferromagnetic, or superparamagnetic depending on the magnetic behavior they show when a magnetic field is applied or removed [101].

Nikitin et al. (2007) [91] designed a biosensor based on nano-sized superparamagnetic particles that exhibit a magnetic behavior under the application of a magnetic field and returning to initial state after its removal. In particular, the detection phase occurs under a magnetic field application at two different frequencies and this allows for the detection of non-linear magnetic material. The sandwich magnetic immunoassays designed for *L. pneumophila* detection were designed on chromatographic nitrocellulose strips, porous filters, and multi-capillary glass structures and were able to detect *L. pneumophila* with an LoD of 10^3^ cells/mL. Cebriàn et al. (2018) [92] applied a magnetic detection-based biosensor for the detection of *Legionella*, using the immunomagnetic separation technique (IMS) and an enzyme immunoassay. The method was able to detect bacterial cells in 1 h with an LoD lower than 100 CFU/L. 

## 7. Mass-Sensitive Measurements

*Surface Acoustic Wave*

Among mass-sensitive measurements, surface acoustic wave (SAW)-based biosensors are extremely useful tools for many purposes thanks to their faster feedback time compared with standard analytical methodologies, as well as their versatilityin bacteria detection. So far, acoustic transducers are implicated in the manufacturing of several kinds of sensors based on SAWs, like shear-horizontal surface acoustic wave (SH-SAW), Rayleigh surface acoustic wave (R-SAW), and Love wave (L-SAW).

Gagliardi et al. (2023) [93] designed a SAW immuno-biosensor for *L. pneumophila* detection in water. The system worked at an ultra-high frequency (740 MHz) and it was functionalized with an anti-*L. pneumophila* antibody to maximize its specificity. Experiments were conducted using suspensions of *L. pneumophila* containing common water contaminants, i.e., *E. coli* and *Enterococcus faecium*, and testing samples from mains water, both clean and contaminated with *L. pneumophila*. The proposed device was able to detect *L. pneumophila* in the range from 1 x 10^6^ to 1 x10^8^ CFU/mL and with an LoD of 2.01 × 10^6^ CFU/mL. 

Finally, Howe et al. (2000) [94] created a dual channel SAW biosensor for *Legionella* and *E. coli* detection in which the direct linkage of the bacteria to the surface of the SAW device is before the addition of the antibodies, comparing it to the standard protocol for SAW in which antibodies were immobilized on the sensor before the bacteria capture. The aforementioned biosensor functionalized with monoclonal *Legionella* antibody and polyclonal *E. coli* H7 antibody showed great sensitivity with an LoD down to 10^6^ cells/mL within 3 h for both bacteria.

## 8. Conclusions

Biosensing technologies show great promise in the detection of *Legionella* in environmental samples, providing rapid, sensitive, and specific methods for isolating and identifying the bacterium. Conventional detection methods, such as culture-based techniques, PCR, and immunoassays, though effective, are often time-consuming and require specialized laboratory equipment. In contrast, biosensors offer the potential for on-site and real-time monitoring, which is crucial for preventing *Legionella* outbreaks and ensuring water safety, ultimately protecting public health.

Early detection is crucial for preventing outbreaks of Legionnaires’ disease and reducing the incidence of disease, ultimately saving lives and reducing healthcare costs associated with treating infected individuals. Real-time monitoring allows for continuous oversight of critical environments, enabling rapid responses to any detected contamination. This can significantly reduce the time between contamination and corrective actions, minimizing the risk of exposure to *Legionella*. Biosensors can be deployed on-site, eliminating the need to transport samples to a laboratory for analysis. This not only speeds up the detection process but also reduces the logistical challenges and costs associated with sample transportation. Finally, the widespread use of biosensors for *Legionella* could contribute to more comprehensive public health surveillance, allowing authorities to track the presence of the bacterium across different environments. Improved surveillance can lead to better understanding and management of *Legionella* risks, informing public health policies and intervention strategies. This data-driven approach can help in identifying hotspots, understanding seasonal trends, and assessing the effectiveness of mitigation measures.

Obviously, each biosensor technology also has its limitations, depending on the specific requirements of sensitivity, selectivity, cost, and environmental conditions. Optical biosensors usually show high sensitivity due to the direct correlation between light signal changes and analyte concentration, as well as high specificity with proper surface functionalization. One of the main limitations is due to potential interference from the sample matrix because if the analyte concentration is too high, it can cause a saturation of the sensor surface, leading to non-linear responses or difficulty in distinguishing small differences in concentration. The second main limitation can be due to improper functionalization, which can lead to reduced sensitivity, poor selectivity, inconsistent signal generation, decreased stability, and difficulties in sensor regeneration and reuse. Thus, ensuring precise and reliable surface functionalization is essential for developing effective and reliable optical biosensors.

Electrochemical bacterial biosensors usually show high sensitivity, especially in detecting bacterial metabolites or redox reactions and good selectivity, often enhanced by using specific biological recognition elements like antibodies or DNA probes. Compared with the other technologies, the main advantages of these biosensors are low cost, simplicity, portability, and suitability for point-of-care testing. On the other hand, they can suffer from contamination of the sensor surface or fouling, and this can be a limitation in their overall performance. In particular, fouling complicates sensor maintenance and shortens operational lifespan, making it a critical issue to address in the design and application of these biosensors. 

Magnetic biosensors show a very high sensitivity, especially in detecting low concentrations of analytes, and high selectivity when using specific magnetic labels. The main advantages are related to their resistance to interference from non-magnetic components (e.g., copper or zinc), and suitability for in vivo applications. Anyway, complexity in sensor fabrication, high cost, and the need for specialized equipment are, at the moment, the main limitations, which make these biosensors less accessible and more challenging to deploy in various applications, especially those requiring low-cost, portable, and easy-to-use solutions. 

Finally, mass-sensitive biosensors show high sensitivity, particularly in detecting small mass changes, and good selectivity, enhanced by functionalizing the sensor surface with specific recognition elements. Anyway, their utility may be constrained by sensitivity to environmental conditions like temperature, humidity, and pressure that can introduce noise and drift, making accurate measurements challenging without stringent environmental control. Moreover, non-specific binding can lead to inaccurate readings, necessitating careful surface functionalization and potentially complex sample preparation. Finally, high costs associated with materials, fabrication, and the need for specialized equipment can limit their widespread adoption, particularly in cost-sensitive applications. 

In conclusion, using biosensors for *Legionella* detection in real-world applications may be fraught with challenges. On the other hand, they have the potential to make a profound impact on public health by enabling the early detection, real-time monitoring, and on-site testing of water systems. The full realization of these benefits depends on overcoming technical and economic challenges to make these biosensors widely accessible and reliable for real-world applications.

## Figures and Tables

**Table 1:** The five main groups of transducers used for biosensors and their subtypes.

Transducers	Subtypes	References
Optical	Colorimetric	Bedrina et al., 2013 [30], Nuthong et al., 2018 [31], Párraga-Niño et al., 2018 [32]
	Photoluminescence	Islam et al., 2022 [33], Islam et al., 2021 [34], Azyzian et al., 2016 [35]
	Chemiluminescence	Yoon et al., 2003 [36], Kober et al., 2018 [37]
	Fluorescence	Martynenko et al., 2019 [38], Wu et al., 2016 [39], Foudeh et al., 2015 [40], Rothenbroker et al., 2021 [41], Eisenreichova et al., 2023 [42], Islam et al., 2020 [43], Delgado-Viscogliosi et al., 2005 [44], Honda et al., 2022 [45], Yamaguchi et al., 2017 [46], Alhogail et al., 2021 [47], Chawich et al., 2022 [48], Curtin et al., 2023 [49], Daneshvar et al., 1999 [50], Lee et al., 2022 [51], Saad et al., 2020 [52]
	MCDA-LFB assay	Jiang et al., 2022 [53]
	Surface plasmon resonance	Oh et al., 2003 [54], Oh et al., 2005 [55], Lin et al., 2007 [56], De Lorenzis et al., 2013 [57], Filion-Côté et al., 2014 [58], Foudeh et al., 2014 [59], Foudeh et al., 2015 [60], Melaine et al., 2017 [61], Meneghello et al., 2017 [62], Karimiravesh et al., 2022 [63], You et al., 2020 [64]
	Photonics	Li et al., 2013 [65]
Electrochemical	Amperometric	Martin et al., 2015 [66], Miranda-Castro et al., 2017 [67], Ezenarro et al., 2020 [68], Mobed et al., 2019 [69]
	Voltammetric	Park et al., 2014 [70], Olabarria et al., 2020 [71], Laribi et al., 2020 [72], Rai et al., 2012 [73], Rai et al., 2012 [74], Miranda-Castro et al., 2009 [75], Miranda-Castro et al., 2008 [76], Mobed et al., 2019 [77], Mobed et al., 2019 [78], Mobed et al., 2019 [79], Park et al., 2010 [80], Lee et al., 2010 [81], Lee et al., 2012 [82], Kim et al., 2012 [83]
	Impedimetric	Lei et al., 2012 [84], Souiri et al., 2014 [85], Sboui et al., 2015 [86], Muhsin et al., 2022 [87], Li et al., 2012 [88], Su et al., 2018 [89], Li et al., 2017 [90]
Magnetic		Nikitin et al., 2007 [91], Cebriàn et al., 2018 [92]
mass-sensitive measurements	Surface acoustic wave	Gagliardi et al., 2023 [93], Howe et al., 2000 [94]

## References

[1] Dagan A., Epstein D., Mahagheh A., Nashashibi J., Geffen Y., Neuberger A., Miller A. (2021). Community-acquired versus nosocomial *Legionella* pneumonia: Factors associated with *Legionella*-related mortality. Eur. J. Clin. Microbiol. Infect. Dis..

[2] Iliadi V., Staykova J., Iliadis S., Konstantinidou I., Sivykh P., Romanidou G., Vardikov D.F., Cassimos D., Konstantinidis T.G. (2022). *Legionella pneumophila*: The Journey from the Environment to the Blood. J. Clin. Med..

[3] Coniglio M.A., Yassin M.H. (2024). Clinical and Environmental Surveillance for the Prevention of Legionellosis. Microorganisms.

[4] Coniglio M.A., Ferrante M., Yassin M.H. (2018). Preventing Healthcare-Associated Legionellosis: Results after 3 Years of Continuous Disinfection of Hot Water with Monochloramine and an Effective Water Safety Plan. Int. J. Environ. Res. Public Health.

[5] Farina C., Cacciabue E., Averara F., Ferri N., Vailati F., Del Castillo G., Serafini A., Fermi B., Doniselli N., Pezzoli F. (2023). Water Safety Plan, Monochloramine Disinfection and Extensive Environmental Sampling Effectively Control Legionella and Other Waterborne Pathogens in Nosocomial Settings: The Ten-Year Experience of an Italian Hospital. Microorganisms.

[6] Sciuto E.L., Laganà P., Filice S., Scalese S., Libertino S., Corso D., Faro G., Coniglio M.A. (2021). Environmental Management of Legionella in Domestic Water Systems: Consolidated and Innovative Approaches for Disinfection Methods and Risk Assesment. Microorganisms.

[7] Filice S., Sciuto E.L., Scalese S., Faro G., Libertino S., Corso D., Timpanaro R.M., Laganà P., Coniglio M.A. (2022). Innovative Antibiofilm Smart Surface against Legionella for Water Systems. Microorganisms.

[8] Fuochi V., Coniglio M.A., Laghi L., Rescifina A., Caruso M., Stivala A., Furneri P.M. (2019). Metabolic Characterization of Supernatants Produced by *Lactobacillus* spp. with in vitro Anti-Legionella Activity. Front. Microbiol..

[9] Martinelli M., Calaresu E., Musumeci R., Giubbi C., Perdoni F., Frugoni S., Castriciano S., Scaturro M., Ricci M.L., Cocuzza C.E. (2021). Evaluation of an Environmental Transport Medium for *Legionella pneumophila* Recovery. Int. J. Environ. Res. Public Health.

[10] Bai L., Yang W., Li Y. (2023). Clinical and Laboratory Diagnosis of Legionella Pneumonia. Diagnostics.

[11] Donohue M.J. (2021). Quantification of Legionella pneumophila by qPCR and culture in tap water with different concentrations of residual disinfectants and heterotrophic bacteria. Sci. Total Environ..

[12] Joly P., Falconnet P.A., André J., Weill N., Reyrolle M., Vandenesch F., Maurin M., Etienne J., Jarraud S. (2006). Quantitative real-time Legionella PCR for environmental water samples: Data interpretation. Appl. Environ. Microbiol..

[13] Sauget M., Richard M., Chassagne S., Hocquet D., Bertrand X., Jeanvoin A. (2023). Validation of quantitative real-time polymerase chain reaction for detection of Legionella pneumophila in hospital water networks. J. Hosp. Infect..

[14] Lu X., Mo Z.Y., Zhao H.B., Yan H., Shi L. (2011). LAMP-based method for a rapid identification of *Legionella* spp. and *Legionella pneumophila*. Appl. Microbiol. Biotechnol..

[15] Caruso G., Coniglio M.A., Laganà P., Fasciana T., Arcoleo G., Arrigo I., Di Carlo P., Palermo M., Giammanco A. (2024). Validation of a Loop-Mediated Isothermal Amplification-Based Kit for the Detection of *Legionella pneumophila* in Environmental Samples According to ISO/TS 12869:2012. Microorganisms.

[16] Soheili M., Nejadmoghaddam M.R., Babashamsi M., Ghasemi J., Jeddi Tehrani M. (2007). Detection of *Legionella pneumophila* by PCR-ELISA method in industrial cooling tower water. Pak. J. Biol. Sci..

[17] Boczek L.A., Tang M., Formal C., Lytle D., Ryu H. (2021). Comparison of two culture methods for the enumeration of *Legionella pneumophila* from potable water samples. J. Water Health.

[18] Walker J.T., McDermott P.J. (2021). Confirming the Presence of *Legionella pneumophila* in Your Water System: A Review of Current Legionella Testing Methods. J. AOAC Int..

[19] Girolamini L., Pascale M.R., Mazzotta M., Spiteri S., Marino F., Salaris S., Grottola A., Orsini M., Cristino S. (2022). Combining Traditional and Molecular Techniques Supports the Discovery of a Novel Legionella Species During Environmental Surveillance in a Healthcare Facility. Front. Microbiol..

[20] Capuano G.E., Corso D., Farina R., Pezzotti Escobar G., Screpis G.A., Coniglio M.A., Libertino S. (2024). Miniaturizable Chemiluminescence System for ATP Detection in Water. Sensors.

[21] Capuano G.E., Farina R., Screpis G., Corso D., Coniglio M.A., Libertino S. (2024). In-Situ Contaminant Detection by Portable and Potentially Real-Time Sensing Systems.

[22] Mobed A., Hasanzadeh M., Agazadeh M., Mokhtarzadeh A., Rezaee M.A., Sadeghi J. (2019). Bioassays: The best alternative for conventional methods in detection of Legionella pneumophila. Int. J. Biol. Macromol..

[23] Kirschner A.K.T. (2016). Review Determination of viable legionellae in engineered water systems: Do we find what we are looking for?. Water Res..

[24] Ngashangva L., Hemdan B.A., El-Liethy M.A., Bachu V., Minteer S.D., Goswami P. (2022). Emerging Bioanalytical Devices and Platforms for Rapid Detection of Pathogens in Environmental Samples. Micromachines.

[25] Page M.J., McKenzie J.E., Bossuyt P.M., Boutron I., Hoffmann T.C., Mulrow C.D., Shamseer L., Tetzlaff J.M., Akl E.A., Brennan S.E. (2021). The PRISMA 2020 statement: An updated guideline for reporting systematic reviews. BMJ.

[26] Mehrotra P. (2016). Biosensors and their applications—A review. J. Oral Biol. Craniofac. Res..

[27] Velusamy V., Arshak K., Korostynska O., Oliwa K., Adley C. (2010). An overview of foodborne pathogen detection: In the perspective of biosensors. Biotechnol. Adv..

[28] Hasanzadeh M.N., Arezoo S., Hassanpour S., Shadjou N., Mokhtarzadeh A., Mohammadi J. (2017). Proline dehydrogenase-entrapped mesoporous magnetic silica nanomaterial for electrochemical biosensing of L-proline in biological fluids. Enzym. Microb. Technol..

[29] Hasanzadeh M., Razmi N., Mokhtarzadeh A., Shadjou N., Mahboob S. (2018). Aptamer based assay of plated-derived grow factor in unprocessed human plasma sample and MCF-7 breast cancer cell lysates using gold nanoparticle supported α-cyclodextrin. Int. J. Biol. Macromol..

[30] Bedrina B., Macián S., Solís I., Fernàndez-Lafuente R., Baldrich E., Rodrìguez G. (2013). Fast immunosensing technique to detect *Legionella pneumophila* in different natural and anthropogenic environments: Comparative and collaborative trials. BMC Microbiol..

[31] Nuthong B., Wilailuckana C., Tavichakorntrakool R., Boonsiri P., Daduang S., Bunyaraksyotin G., Suphan O., Daduang J. (2018). One step for *Legionella pneumophila* detection in environmental samples by DNA-gold nanoparticle probe. J. Appl. Microbiol..

[32] Párraga-Niño N., Quero S., Ventós-Alfonso A., Uria N., Castillo-Fernandez O., Ezenarro J.J., Muñoz F.-X., Garcia-Nuñez M., Sabrià M. (2018). New system for the detection of *Legionella pneumophila* in water samples. Talanta.

[33] Islam M.A., Hassen W.M., Ishika I., Tayabali A.F., Dubowski J.J. (2022). Selective Detection of *Legionella pneumophila* Serogroup 1 and 5 with a Digital Photocorrosion Biosensor Using Antimicrobial Peptide-Antibody Sandwich Strategy. Biosensors.

[34] Islam M.A., Hassen W.M., Tayabali A.F., Dubowski J.J. (2021). Short Ligand, Cysteine-Modified Warnericin RK Antimicrobial Peptides Favor Highly Sensitive Detection of *Legionella pneumophila*. ACS Omega.

[35] Aziziyan M.R., Hassen W.M., Morris D., Frost E.H., Dubowski J.J. (2016). Photonic biosensor based on photocorrosion of GaAs/AlGaAs quantum heterostructures for detection of *Legionella pneumophila*. Biointerphases.

[36] Yoon C.H., Cho J.H., Oh H.I., Kim M.J., Lee C.W., Choi J.W., Paek S.H. (2003). Development of a membrane strip immunosensor utilizing ruthenium as an electro-chemiluminescent signal generator. Biosens. Bioelectron..

[37] Kober C., Niessner R., Seidel M. (2018). Quantification of viable and non-viable *Legionella* spp. by heterogeneous asymmetric recombinase polymerase amplification (haRPA) on a flow-based chemiluminescence microarray. Biosens. Bioelectron..

[38] Martynenko I.V., Kusić D., Weigert F., Stafford S., Donnelly F.C., Evstigneev R., Gromova Y., Baranov A.V., Rühle B., Kunte H.-J. (2019). Magneto-Fluorescent Microbeads for Bacteria Detection Constructed from Superparamagnetic Fe_3_O_4_ Nanoparticles and AIS/ZnS Quantum Dots. Anal. Chem..

[39] Wu T.Y., Su Y.Y., Shu W.H., Mercado A.T., Wang S.K., Hsu L.Y., Tsai Y.F., Chen C.Y. (2016). A novel sensitive pathogen detection system based on Microbead Quantum Dot System. Biosens. Bioelectron..

[40] Foudeh A.M., Brassard D., Tabrizian M., Veres T. (2015). Rapid and multiplex detection of Legionella’s RNA using digital microfluidics. Lab Chip.

[41] Rothenbroker M., McConnell E.M., Gu J., Urbanus M.L., Samani S.E., Ensminger A.W., Filipe C.D.M., Li Y. (2021). Selection and Characterization of an RNA-Cleaving DNAzyme Activated by *Legionella pneumophila*. Angew. Chem. Int. Ed. Engl..

[42] Eisenreichova A., Humpolickova J., Różycki B., Boura E., Koukalova A. (2023). Effects of biophysical membrane properties on recognition of phosphatidylserine, or phosphatidylinositol 4-phosphate by lipid biosensors LactC2, or P4M. Biochimie.

[43] Islam M.A., Hassen W.M., Tayabali A.F., Dubowski J.J. (2020). Antimicrobial warnericin RK peptide functionalized GaAs/AlGaAs biosensor for highly sensitive and selective detection of *Legionella pneumophila*. Biochem. Eng. J..

[44] Delgado-Viscogliosi P., Simonart T., Parent V., Marchand G., Dobbelaere M., Pierlot E., Pierzo V., Menard-Szczebara F., Gaudard-Ferveur E., Delabre K. (2005). Rapid method for enumeration of viable *Legionella pneumophila* and other *Legionella* spp. in water. Appl. Environ. Microbiol..

[45] Honda Y., Ichikawa R., Choi Y.J., Murakami K., Takahashi K., Noda T., Sawada K., Ishii H., Machida K., Ito H. (2022). Detection system for Legionella bacteria using photogate-type optical sensor. J. Appl. Phys..

[46] Yamaguchi N., Tokunaga Y., Goto S., Fujii Y., Banno F., Edagawa A. (2017). Rapid on-site monitoring of *Legionella pneumophila* in cooling tower water using a portable microfluidic system. Sci. Rep..

[47] Alhogail S., Chinnappan R., Alrifai M., Suaifan G.A.R.Y., Bikker F.J., Kaman W.E., Weber K., Cialla-May D., Popp J., Alfageeh M.B. (2021). Simple and rapid peptide nanoprobe biosensor for the detection of Legionellaceae. Analyst.

[48] Chawich J., Hassen W.M., Singh A., Marquez D.T., DeRosa M.C., Dubowski J.J. (2022). Polymer Brushes on GaAs and GaAs/AlGaAs Nanoheterostructures: A Promising Platform for Attractive Detection of *Legionella pneumophila*. ACS Omega.

[49] Curtin K., Wang J., Fike B.J., Binkley B., Li P. (2023). A 3D printed microfluidic device for scalable multiplexed CRISPR-cas12a biosensing. Biomed. Microdevices.

[50] Daneshvar M.I., Peralta J.M., Casay G.A., Narayanan N., Evans L., Patonay G., Strekowski L. (1999). Detection of biomolecules in the near-infrared spectral region via a fiber-optic immunosensor. J. Immunol. Methods.

[51] Lee H.M., Choi D.W., Kim S., Lee A., Kim M., JinRoh Y., Ho Jo Y., YeonCho H., Lee H.J., Lee S.R. (2022). Biosensor-Linked Immunosorbent Assay for the Quantification of Methionine Oxidation in Target Proteins. ACS Sens..

[52] Saad M., Chinerman D., Tabrizian M., Faucher S.P. (2020). Identification of two aptamers binding to *Legionella pneumophila* with high affinity and specificity. Sci. Rep..

[53] Jiang L., Gu R., Li X., Song M., Huang X., Mu D. (2022). Multiple Cross Displacement Amplification Coupled with Lateral Flow Biosensor (MCDA-LFB) for rapid detection of *Legionella pneumophila*. BMC Microbiol..

[54] Oh B.K., Kim Y.K., Lee W., Bae Y.M., Lee W.H., Choi J.W. (2003). Immunosensor for detection of *Legionella pneumophila* using surface plasmon resonance. Biosens. Bioelectron..

[55] Oh B.K., Lee W., Chun B.S., Bae Y.M., Lee W.H., Choi J.W. (2005). The fabrication of protein chip based on surface plasmon resonance for detection of pathogens. Biosens. Bioelectron..

[56] Lin H.Y., Tsao Y.C., Tsai W.H., Yang Y.W., Yan T.R., Sheu B.C. (2007). Development and application of side-polished fiber immunosensor based on surface plasmon resonance for the detection of *Legionella pneumophila* with halogens light and 850 nm-LED. Sens. Actuators A Phys..

[57] De Lorenzis E., Manera M.G., Montagna G., Cimaglia F., Chiesa M., Poltronieri P., Santino A., Rella R. (2013). SPR based immunosensor for detection of *Legionella pneumophila* in water samples. Opt. Commun..

[58] Filion-Côté S., Roche P.J.R., Foudeh A.M., Tabrizian M., Kirk A.G. (2014). Design and analysis of a spectro-angular surface plasmon resonance biosensor operating in the visible spectrum. Rev. Sci. Instrum..

[59] Foudeh A.M., Daoud J.T., Faucher S.P., Veres T., Tabrizian M. (2014). Sub-femtomole detection of 16s rRNA from *Legionella pneumophila* using surface plasmon resonance imaging. Biosens. Bioelectron..

[60] Foudeh A.M., Trigui H., Mendis N., Faucher S.P., Veres T., Tabrizian M. (2015). Rapid and specific SPRi detection of *L. pneumophila* in complex environmental water samples. Anal. Bioanal. Chem..

[61] Melaine F., Saad M., Faucher S., Tabrizian M. (2017). Selective and High Dynamic Range Assay Format for Multiplex Detection of Pathogenic *Pseudomonas aeruginosa*, *Salmonella typhimurium*, and *Legionella pneumophila* RNAs Using Surface Plasmon Resonance Imaging. Anal. Chem..

[62] Meneghello A., Sonato A., Ruffato G., Zacco G., Romanato F. (2017). A novel high sensitive surface plasmon resonance *Legionella pneumophila* sensing platform. Sens. Actuators B Chem..

[63] Karimiravesh R., Mohabat Mobarez A., Behmanesh M., Nikkhah M., Talebi Bezmin Abadi A., Esmaeilli S. (2022). Design of an optical nano biosensor for detection of *Legionella pneumophila* in water samples. Iran. J. Microbiol..

[64] You Y., Lim S., Gunasekaran S. (2020). Streptavidin-Coated Au Nanoparticles Coupled with Biotinylated Antibody-Based Bifunctional Linkers as Plasmon-Enhanced Immunobiosensors. ACS Appl. Nano Mater..

[65] Li N., Cheng X.R., Brahmendra A., Prashar A., Endo T., Guyard C., Terebiznik M., Kerman K. (2013). Photoniccrystals on copolymer film for bacteriadetection. Biosens. Bioelectron..

[66] Martín M., Salazar P., Jiménez C., Lecuona M., Ramos M.J., Ode J., Alcoba J., Roche R., Villalonga R., Campuzano S. (2015). Rapid *Legionella pneumophila* determination based on a disposable core-shell Fe_3_O_4_@poly(dopamine) magnetic nanoparticles immunoplatform. Anal. Chim. Acta.

[67] Miranda-Castro R., Sánchez-Salcedo R., Suárez-Álvarez B., de-los-Santos-Álvarez N., Miranda-Ordieres A.J., Lobo-Castañón M.J. (2017). Thioaromatic DNA monolayers for target-amplification-free electrochemical sensing of environmental pathogenic bacteria. Bionsens. Bioelectron..

[68] Ezenarro J.J., Párraga-Niño N., Sabrià M., Del Campo F.J., Muñoz-Pascual F.-X., Mas J., Uria N. (2020). Rapid Detection of *Legionella pneumophila* in Drinking Water, Based on Filter Immunoassay and Chronoamperometric Measurement. Biosensors.

[69] Mobed A., Hasanzadeh M., Shadjou N., Hassanpour S., Saadati A., Agazadeh M. (2019). Immobilization of ssDNA on the surface of silver nanoparticles-graphene quantum dots modified by gold nanoparticles towards biosensing of microorganism. Microchem. J..

[70] Park J., You X., Jang Y., Nam Y., Kim M.J., Min N.K., Pak J.J. (2014). ZnO nanorod matrix based electrochemical immunosensors for sensitivity enhanced detection of *Legionella pneumophila*. Sens. Actuators B Chem..

[71] Olabarria G., Eletxigerra U., Rodriguez I., Bilbao A., Berganza J., Merino S. (2020). Highly sensitive and fast *Legionella* spp. in situ detection based on a loop mediated isothermal amplification technique combined to an electrochemical transduction system. Talanta.

[72] Laribi A., Allegra S., Souiri M., Mzoughi R., Othmane A., Girardot F. (2020). *Legionella pneumophila* sg1-sensing signal enhancement using a novel electrochemical immunosensor in dynamic detection mode. Talanta.

[73] Rai V., Deng J., Toh C.-S. (2012). Electrochemical nanoporous alumina membrane-based label-free DNA biosensor for the detection of *Legionella* sp.. Talanta.

[74] Rai V., Nyine Y.T., Hapuarachchi H.C., Yap H.M., Ng L.C., Toh C.S. (2012). Electrochemically amplified molecular beacon biosensor for ultrasensitive DNA sequence-specific detection of *Legionella* sp.. Biosens. Bioelectron..

[75] Miranda-Castro R., de-los-Santos-Álvarez N., Lobo-Castañón M.J., Miranda-Ordieres A.J., Tuñón-Blanco P. (2009). PCR-coupledelectrochemical sensing of *Legionella pneumophila*. Biosens. Bioelectron..

[76] Miranda-Castro R., Lobo-Castañón M.J., Miranda-Ordieres A., Tuñón-Blanco P. (2008). Stem-Loop DNA Probes for the Voltammetric Determination of *Legionella pneumophila* on Disposable Screen-Printed Gold Electrodes. Electroanalysis.

[77] Mobed A., Hasanzadeh M., Babaie P., Aghazadeh M., Mokhtarzadeh A., Ahangarzadeh Rezaee M. (2019). Cetyltrimethyl ammonium bromide modified gold nanostructure supported by chitosan as a novel scaffold for immobilization of DNA and ultra-sensitive bioassay of *Legionella pneumophila*. Microchem. J..

[78] Mobed A., Hasanzadeh M., Hassanpour S., Saadati A., Agazadeh M., Mokhtarzadeh A. (2019). An innovative nucleic acid- based biosensor toward detection of *Legionella pneumophila* using DNA immobilization and hybridization: A novel genosensor. Microchem. J..

[79] Mobed A., Hasanzadeh M., Babaie P., Agazadeh M., Mokhtarzadeh A., Ahangarzadeh Rezaee M. (2019). DNA-based bioassay of legionella pneumonia pathogen using gold nanostructure: A new platform for diagnosis of legionellosis. Int. J. Biol. Macromol..

[80] Park E.J., Lee J.-Y., Kim J.H., Lee C.J., Kim H.S., Min N.K. (2010). Investigation of plasma-functionalized multiwalled carbon nanotube film and its application of DNA sensor for *Legionella pneumophila* detection. Talanta.

[81] Lee J.Y., Park E.J., Kim J.H., Kim S.G., Lee C.J., Kim M.J., Min N.K. (2010). O_2_ plasma patterning of p-type MWCNT and its application to immunosensor. Thin Solid. Films.

[82] Lee J.Y., Jin J.H., Kim J.H., Kim M.J., Lee C.J., Min N.K. (2012). Plasma-Activated Carbon Nanotube-Based High Sensitivity Immunosensors for Monitoring *Legionella pneumophila* by Direct Detection of Maltose Binding Protein Peptidoglycan-Associated Lipoprotein (MBP-PAL). Biotechnol. Bioeng..

[83] Kim J.H., Lee J.-Y., Jin J.-H., Park C.W., Lee C.J., Min N.K. (2012). A fully microfabricated carbon nanotube three-electrode system on glass substrate for miniaturized electrochemical biosensors. Biomed. Microdevices.

[84] Lei K.F., Leung P.H.M. (2012). Microelectrode array biosensor for the detection of *Legionella pneumophila*. Microelectron. Eng..

[85] Souiri M., Blel N., Sboui D., Mhamdi L., Epalle T., Mzoughi R., Riffard S., Othmane A. (2014). AFM, CLSM and EIS characterization of the immobilization of antibodies on indium-tin oxide electrode and their capture of *Legionella pneumophila*. Talanta.

[86] Sboui D., Souiri M., Reynaud S., Palle S., Ismail M.B., Epalle T., Mzoughi R., Girardot F., Allegra S., Riffard S. (2015). Characterisation of electrochemical immunosensor for detection of viable not-culturable forms of *Legionellla pneumophila* in water samples. Chem. Pap..

[87] Muhsin S.A., Al-Amidie M., Shen Z., Mlaji Z., Liu J., Abdullah A., El-Dweik M., Zhang S., Almasri M. (2022). A microfluidic biosensor for rapid simultaneous detection of waterborne pathogens. Biosens. Bioelectron..

[88] Li N., Brahmendra A., Veloso A.J., Prashar A., Cheng X.R., Hung V.W.S., Guyard C., Terebiznik M., Kerman K. (2012). Disposable Immunochips for the Detection of *Legionella pneumophila* Using Electrochemical Impedance Spectroscopy. Anal. Chem..

[89] Su H., Li S., Terebiznik M., Guyard C., Kerman K. (2018). Biosensors for the Detection of Interaction between *Legionella pneumophila* Collagen-Like Protein and Glycosaminoglycans. Sensors.

[90] Li Q., Zhao C., Zheng Z., Weng S., Chen Q., Liu Q., Lin X. (2017). A signal-off double probes electrochemical DNA sensor for the simultaneous detection of *Legionella* and *Legionella pneumophila*. J. Electroanal. Chem..

[91] Nikitin P.I., Vetoshko P.M., Ksenevich T.I. (2007). New type of biosensor based on magnetic nanoparticle detection. J. Magn. Magn. Mater..

[92] Cebrián F., Montero J.C., Fernández P.J. (2018). New approach to environmental investigation of an explosive legionnaires’ disease outbreak in Spain: Early identification of potential risk sources by rapid *Legionella* spp. immunosensing technique. BMC Infect. Dis..

[93] Gagliardi M., Agostini M., Lunardelli F., Lamanna L., Miranda A., Bazzichi A., Luminare A.G., Cervelli F., Gambineri F., Totaro M. (2023). Surface acoustic wave-based lab-on-a-chip for the fast detection of *Legionella pneumophila* in water. Sens. Actuators B Chem..

[94] Howe E., Harding G. (2000). A comparison of protocols for the optimisation of detection of bacteria using a surface acoustic wave (SAW) biosensor. Biosens. Bioelectron..

[95] Deusenbery C., Wang Y., Shukla A. (2021). Recent Innovations in Bacterial Infection Detection and Treatment. ACS Infect. Dis..

[96] Farina R., Scalese S., Corso D., Capuano G.E., Screpis G.A., Coniglio M.A., Condorelli G.G., Libertino S. (2024). Chronoamperometric Ammonium Ion Detection in Water via Conductive Polymers and Gold Nanoparticles. Molecules.

[97] Bard A.J., Faulkner L.R. (2000). Electrochemical Methods: Fundamentals and Applications.

[98] Farina R., D’Arrigo G., Alberti A., Scalese S., Capuano G.E., Corso D., Screpis G.A., Coniglio M.A., Condorelli G.G., Libertino S. (2024). Copper Micro-Flowers for Electrocatalytic Sensing of Nitrate Ions in Water. Sensors.

[99] Scott K., Scott K., Yu E.H. (2016). Electrochemical principles and characterization of bioelectrochemical systems. Microbial Electrochemical and Fuel Cells.

[100] Furst A.L., Francis M.B. (2019). Impedance-Based Detection of Bacteria. Chem. Rev..

[101] Chen Y.-T., Kolhatkar A.G., Zenasni O., Xu S., Lee T.R. (2017). Biosensing Using Magnetic Particle Detection Techniques. Sensors.

